# Production of Squalene in *Synechocystis* sp. PCC 6803

**DOI:** 10.1371/journal.pone.0090270

**Published:** 2014-03-13

**Authors:** Elias Englund, Bagmi Pattanaik, Sarojini Jayantha K. Ubhayasekera, Karin Stensjö, Jonas Bergquist, Pia Lindberg

**Affiliations:** 1 Microbial Chemistry, Department of Chemistry, Ångström and Science for Life Laboratory, Uppsala University, Uppsala, Sweden; 2 Analytical Chemistry, Department of Chemistry, BMC and Science for Life Laboratory, Uppsala, Sweden; University of New South Wales, Australia

## Abstract

In recent years, there has been an increased interest in the research and development of sustainable alternatives to fossil fuels. Using photosynthetic microorganisms to produce such alternatives is advantageous, since they can achieve direct conversion of carbon dioxide from the atmosphere into the desired product, using sunlight as the energy source. Squalene is a naturally occurring 30-carbon isoprenoid, which has commercial use in cosmetics and in vaccines. If it could be produced sustainably on a large scale, it could also be used instead of petroleum as a raw material for fuels and as feedstock for the chemical industry. The unicellular cyanobacterium *Synechocystis* PCC 6803 possesses a gene, *slr2089*, predicted to encode squalene hopene cyclase (Shc), an enzyme converting squalene into hopene, the substrate for forming hopanoids. Through inactivation of *slr2089* (*shc*), we explored the possibility to produce squalene using cyanobacteria. The inactivation led to accumulation of squalene, to a level over 70 times higher than in wild type cells, reaching 0.67 mg OD_750_^−1^ L^−1^. We did not observe any significant growth deficiency in the Δ*shc* strain compared to the wild type *Synechocystis*, even at high light conditions, suggesting that the observed squalene accumulation was not detrimental to growth, and that formation of hopene by Shc is not crucial for growth under normal conditions, nor for high-light stress tolerance. Effects of different light intensities and growth stages on squalene accumulation in the Δ*shc* strain were investigated. We also identified a gene, *sll0513*, as a putative squalene synthase in *Synechocystis*, and verified its function by inactivation. In this work, we show that it is possible to use the cyanobacterium *Synechocystis* to generate squalene, a hydrocarbon of commercial interest and a potential biofuel. We also report the first identification of a squalene hopene cyclase, and the second identification of squalene synthase, in cyanobacteria.

## Introduction

**Figure 1:**
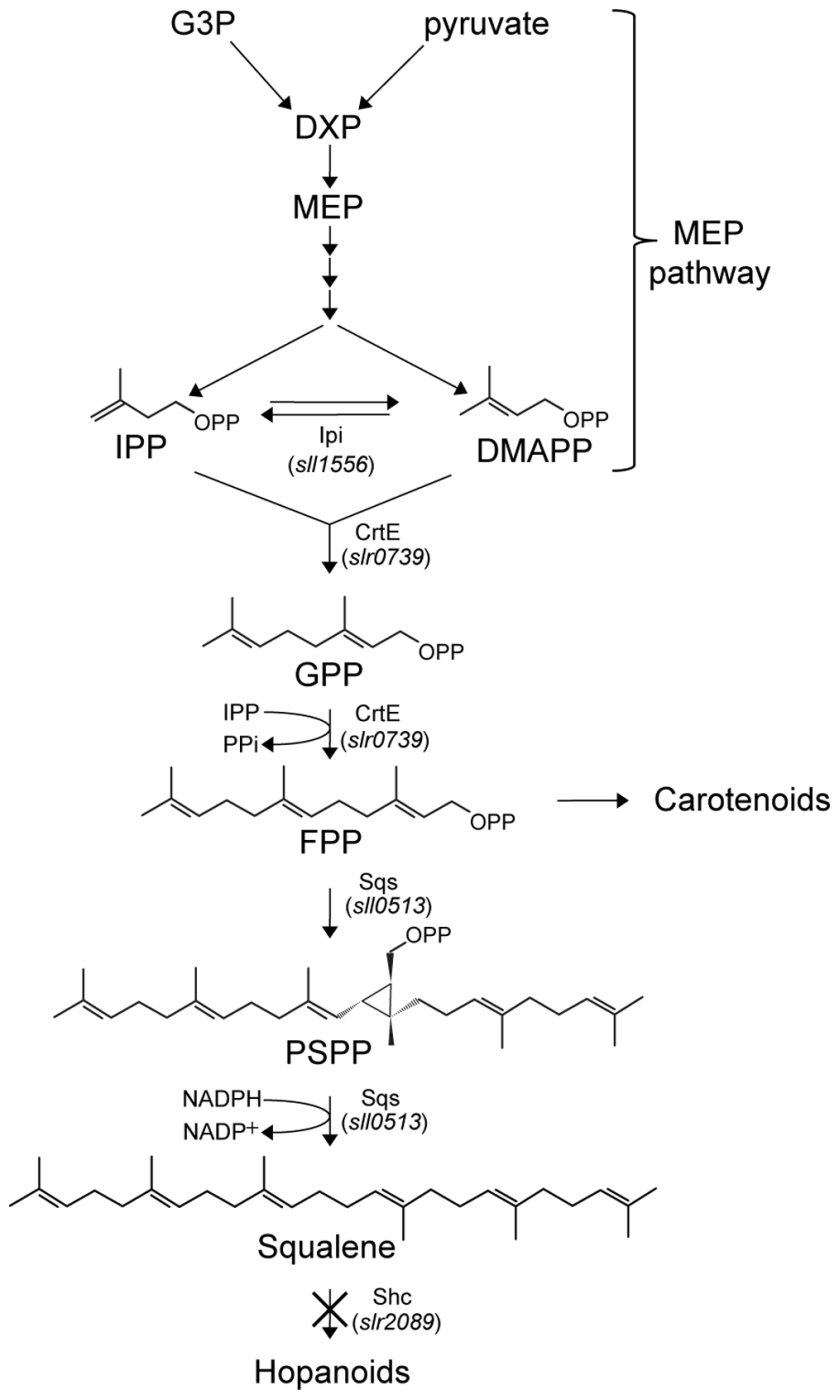
Squalene biosynthesis pathway through the MEP-pathway in *Synechocystis*. Abbreviations used: G3P = glyceraldehyde 3-phosphate; DXP = deoxyxylulose 5-phosphate; MEP = methylerythritol 4-phosphate; IPP = isopentenyl diphosphate; DMAPP = dimethylallyl diphosphate; GPP = geranyl diphosphate; FPP = farnesyl diphosphate; PSPP = presqualene diphosphate. For enzymes: Ipi = isopentenyl diphosphate delta isomerase; CrtE = geranylgeranyl pyrophosphate synthase; Sqs = squalene synthase; Shc = squalene hopene cyclase. Based on data from [3], [5], [6], [7], [8].

Isoprenoids, also called terpenoids, are a large family of compounds including carotenoids, tocopherol, phytol, sterols and hormones. In most prokaryotes, in algae, and in plant plastids, isoprenoids can be produced via the methyl-erythritol-4-phosphate (MEP) pathway ([1], see Fig. 1). This pathway was first characterized in *Escherichia coli*, and uses pyruvate and glyceraldehyde 3-phosphate as substrates [2] to form, in several steps, the products isopentenyl diphosphate (IPP) and dimethylallyl diphosphate (DMAPP) (see Fig. 1). IPP and DMAPP are the building blocks for all other isoprenoids, some of which have useful commercial applications in nutrition, medicine, chemistry, and potentially as biofuels.

The sequenced genome of the unicellular cyanobacterium *Synechocystis* sp. PCC 6803 (from here on referred to as *Synechocystis*) [3] contains all the genes needed to encode the enzymes involved in the MEP pathway in *E. coli* [4] (see Fig. 1). Only a few studies have investigated this pathway in cyanobacteria [5], [6], [7], [8], despite the fact that it is the origin of many interesting and potentially useful compounds. Recently, it was shown that *Synechocystis* can be used for production of isoprene, a small (C_5_H_8_) volatile hydrocarbon [9], and for photosynthetic generation of *β*-phellandrene (C_10_H_16_), an essential oil [10], as a side reaction to the isoprenoid biosynthesis. We are now interested in investigating if it is possible to use cyanobacteria for generation of longer-chain isoprenoid hydrocarbons. Using cyanobacteria for direct production of a biofuel is advantageous, since they can grow photosynthetically on minimal media, fixing carbon dioxide from air and using sunlight as an energy source to generate the product.

The isoprenoid squalene is a 30-carbon pure hydrocarbon, the formation of which is catalyzed by the enzyme squalene synthase. Squalene synthase performs a two-step reaction, where two molecules of farnesyl-diphosphate (FPP) are first combined to form presqualene diphosphate (PSPP), which is subsequently converted into squalene, in a NADPH-dependent step ([11], Fig. 1). The mechanism of this reaction has been thoroughly investigated, primarily in eukaryotes [12], [13]. Today, commercial uses of squalene include as an ingredient in cosmetic products and in vaccines, as an additive in some adjuvant formulations, but if it could be produced sustainably and in large quantities, it could also be used as a raw material for biofuels and as feedstock for the chemical industry.

In a wide range of bacteria, squalene is used as the substrate for formation of hopene, a complex pentacyclic hydrocarbon which is further modified to form hopanoids [14], [15]. The enzyme catalyzing the formation of hopene from squalene, squalene hopene cyclase (Shc) has been characterized in a number of organisms [16], [17], [18], [19], [20], and the structure of Shc from *Alicyclobacillus acidocaldarius* has been determined [21]. Presence of hopanoids in the outer membrane and in the thylakoid membranes have been observed in the cyanobacterium *Synechocystis* PCC 7614 [22], however, to our knowledge, no investigation has yet been carried out regarding production of squalene, or its use in the cell by the action of squalene hopene cyclase, in cyanobacteria.

In this study, we have generated a squalene-producing strain of the cyanobacterium *Synechocystis*. This was achieved by inactivating the gene *slr2089*, putatively encoding the enzyme squalene hopene cyclase (Shc). Inactivation of this single gene leads to accumulation of squalene in the cell. In addition, we identified the gene encoding squalene synthase in *Synechocystis*.

## Results and Discussion

### Genes in *Synechocystis* Putatively Involved in Synthesis and Use of Squalene

In the genome of *Synechocystis* [3], it is possible to identify all the genes encoding the MEP pathway in *E. coli*, as has previously been noted [4]. Since the aim of this study was to investigate production of squalene in *Synechocystis*, we looked further for genes that could be involved in synthesis and utilization of squalene in this strain.

One gene, *slr2089*, can be identified as likely encoding squalene hopene cyclase (Shc) in *Synechocystis*. The putative Shc amino acid sequence is homologous to other Shcs in the databases, with identity/similarity of 43%/58% to the structurally known Shc from *A. acidocaldarius* (PDB: 2SQC_A), and contains known conserved motifs such as the catalytic aspartate identified in *A. acidocaldarius* [21], a DXDD motif in the active site cavity important for the activity of the enzyme [23], and repeated QW-motifs [24]. It exhibits the highest similarities to other putative Shcs in cyanobacteria (>60% identity, >70% similarity). However, *shc* does not appear to be universally present in cyanobacteria. Based on cyanobacterial genome sequences available in the Cyanobase (http://genome.kazusa.or.jp/cyanobase/) and JGI (http://img.jgi.doe.gov/cgi-bin/w/main.cgi) databases, *shc* is present in about 45% of the sequenced strains (data not shown). This is in agreement with data for other organisms, where estimates of the distribution of hopanoid biosynthesis range from 4% of microorganisms in the oceans [25] to 50% of a set of cultured strains [26]. It is clear that the presence of *shc* and hopanoid biosynthesis is not universal and may be an unusual trait in the global microbiome.

A blast search for squalene synthase in the *Synechocystis* genome resulted in identification of the gene *sll0513*, annotated as encoding a hypothetical protein. The amino acid sequence of the *sll0513* gene product (GenBank accession no BAA10820) shows similarities with squalene synthases in other organisms (*e.g.* 52%/72% identity/similarity to farnesyl-diphosphate farnesyltransferase from *Bacillus megaterium* WSH-002, 31%/42% identity/similarity to Sqs from *Botryococcus braunii* (GenBank accession no AAF20201.1), 26%/42% identity/similarity to Sqs from *Saccharomyces cerevisiae* (ERG9, GenBank accession no AAA34597.1)), with the highest similarities to other cyanobacterial sequences (>60% identity, >80% similarity to a number of putative cyanobacterial squalene synthases). In the cyanobacterium *Thermosynechococcus elongatus* BP-1, squalene synthase, encoded by *sqs,* has been experimentally verified [27]. However, there are substantial differences between *sll0513* and *sqs* in *T. elongatus*; *sll0513* encodes a 277 aa protein, whereas Sqs in *T. elongatus* is 359 aa, and their mutual identity/similarity is 29.5%/41.1%. *sll0513* is also similar to phytoene synthases, however, in *Synechocystis* there is another gene, *crtB*, which has been shown to encode phytoene synthase [28]. Furthermore, the deduced amino acid sequence of *sll0513* contains previously identified conserved domains common to squalene synthases [11], [12], [29], including a putative NADPH binding site not present in phytoene synthase [30] (data not shown).

The substrate for the squalene synthase, farnesyl diphosphate, is formed through linking of one molecule of IPP and one molecule of DMAPP to form geranyl-diphosphate, followed by addition of another molecule of IPP. In the *Synechocystis* genome, there is one gene, *crtE* (*slr0739*), annotated as geranylgeranyl diphosphate synthase, which is likely to encode the enzyme that performs these steps.

Figure 1 summarizes the proposed pathway for squalene synthesis and utilization in *Synechocystis*.

### Inactivation of *shc* in *Synechocystis* and Detection of an *shc* Transcript

**Figure 2:**
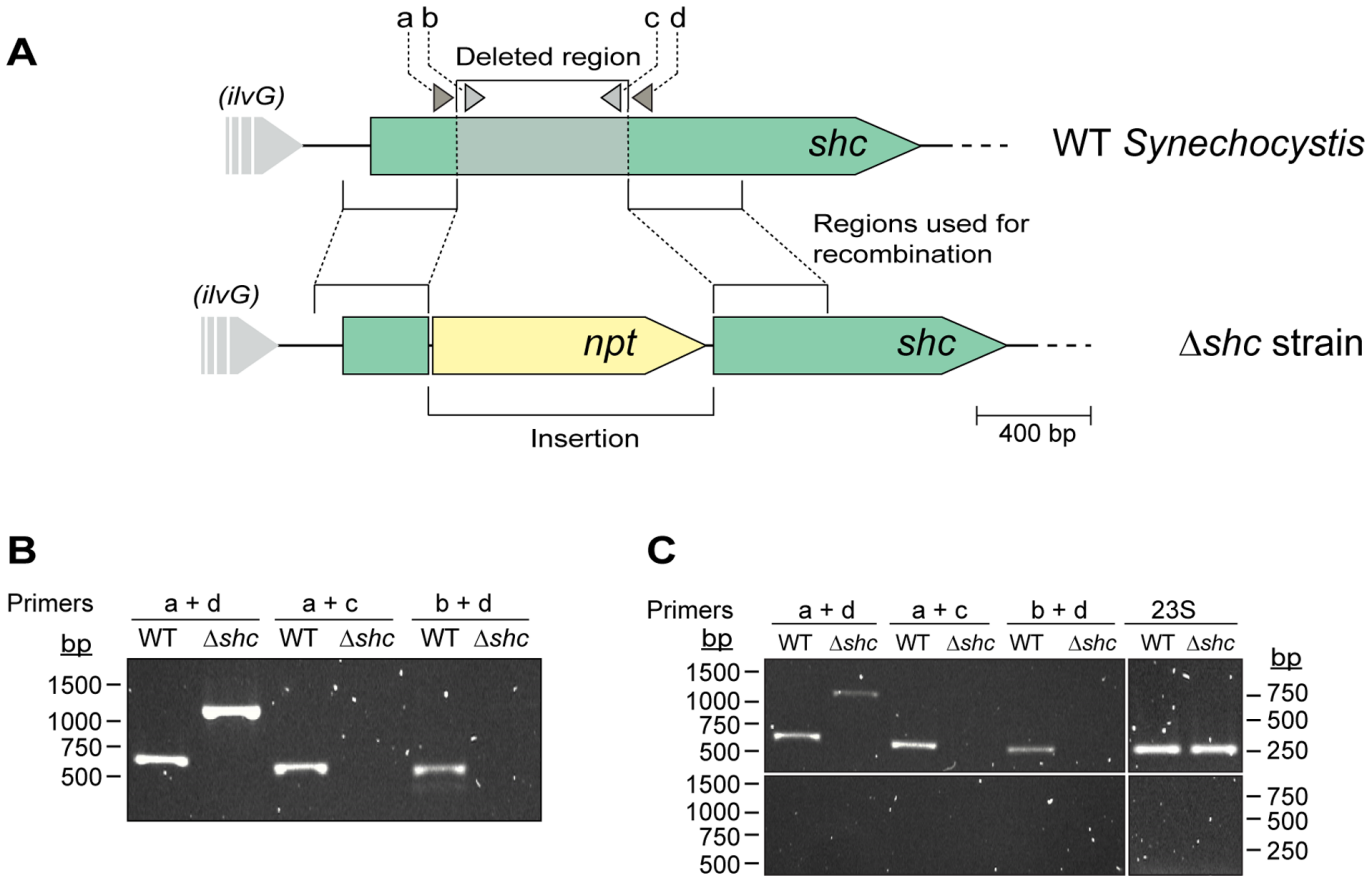
Knock-out strategy and screening of mutant. (A) Schematic overview of the knock-out strategy for *shc*. A neomycin (*npt*) cassette is inserted into *shc* by homologous recombination thereby deleting a part of *shc*. (B) PCR screening of genomic DNA for checking segregation of the mutant compared to wild type. Primers used for screening were a-d where a = shc_usR_as; b = shc_middle_F; c = shc_middle_R; d = shc_dsF_as. (C) RT-PCR of Δ*shc* and wild type cDNA using reverse transcriptase (top) and without (bottom) as negative controls. Primers amplifying 23S were used as a control of equal loading of RNA.

The gene *slr2089* in the *Synechocystis* genome [3], putatively encoding Shc, was inactivated by replacing a 606 bp region of the gene with a neomycin resistance cassette (Fig. 2A). The inactivated gene construct was transferred to *Synechocystis* via natural transformation to generate a Δ*shc* strain. Transformants were isolated by selection with appropriate antibiotics, and replacement of the wild type copy of the gene with the inactivated version was confirmed by PCR. Expected PCR fragments were amplified from the successful Δ*shc* inactivation strains (Fig. 2B). Furthermore, RNA was isolated from the wild type and Δ*shc* strains and used for detection of *shc* transcript in RT-PCR experiments. Transcripts could be detected in both wild type and Δ*shc* cells; however, amplification of transcripts from the deleted region resulted in a product only from the wild type strain (Fig. 2C, left panels). This shows that the gene is actively transcribed under standard photoautotrophic growth conditions in the wild type *Synechocystis*, and that while transcription of the gene is still active in the Δ*shc* strain, there is no intact transcript present. Amplification of 23S cDNA was used as a positive control (Fig. 2C, right panels).

Sequencing of genomic DNA from the Δ*shc* strain was done to further verify the inactivation, and the results reaffirmed that the antibiotic cassette was positioned inside the *shc* gene (data not shown).

### Extraction and Detection of Squalene in the Δ*shc* and Wild Type Strains

**Figure 3:**
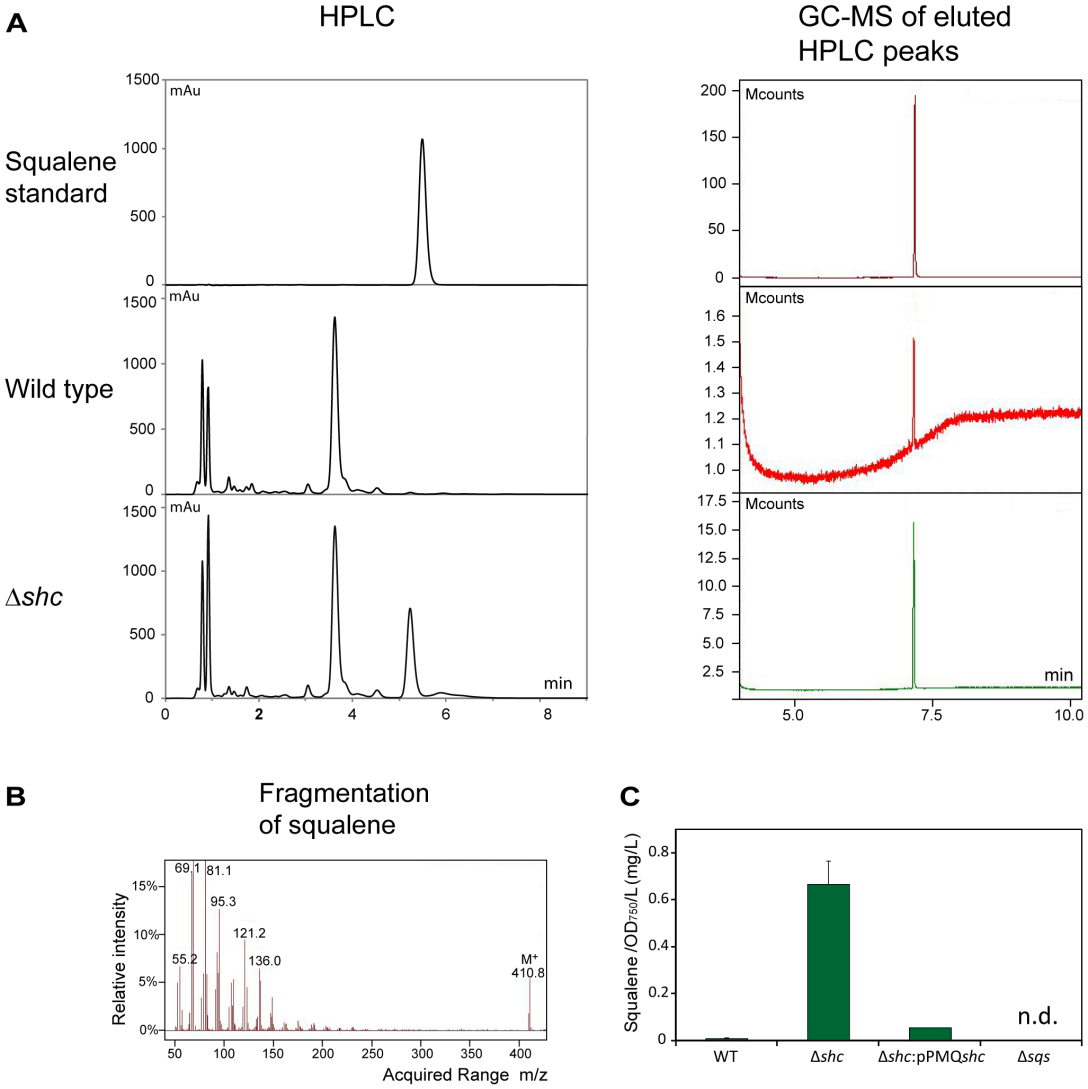
Analysis of squalene accumulation. Squalene was extracted from wild type and Δ*shc* cultures and the extracts analyzed by HPLC using a squalene standard for identification and quantification of the squalene peak. The identity of the squalene peak was confirmed using GC-MS. (A) HPLC chromatograms (left panels) and GC-MS chromatograms (right panels) for the detection of squalene. A squalene standard (top) was compared with cell extracts from wt (middle) and Δ*shc* mutant (bottom). (B) Fragmentation of squalene standard detected using GC-MS (C) Comparison of squalene content between wild type, Δ*shc*, Δ*shc*:pPMQ*shc* complemented strain, and Δ*sqs* cells. n.d. = no squalene detected.

After inactivation of *shc*, we hypothesized that squalene may be accumulating in the cells. To investigate this possibility, a method for extraction and detection of squalene from *Synechocystis* was developed, based on the method for total lipid extraction by Bligh and Dyer [31] (see Materials and Methods for details). Total lipids were extracted from cultured cells using methanol and chloroform, the resulting lipids were dissolved in heptane, and squalene content was determined using HPLC (Fig. 3A), by comparison to a commercially available squalene standard. To further verify the identity of the squalene peak observed by HPLC of cyanobacterial lipid extracts, the peak was eluted and analyzed by GC-MS (Fig. 3A). In both wild type and Δ*shc*, it was found that the eluted peak exhibited the correct fragmentation as compared to a squalene standard (Fig. 3B). However, only minimal amounts of squalene could be detected in the wild type, confirming our results from HPLC. *Synechocystis* cells with *shc* inactivated grown to stationary phase had a squalene content of 0.67±0.102 mg OD_750_^−1^ L^−1^ while the wild type contained 0.0093±0.0031 mg OD_750_^−1^ L^−1^ (Fig. 3C). Thus, squalene accumulated in the Δ*shc* strain to a level more than 70 times the level in the wild type. This result, together with the RT-PCR results showing active transcription of *slr2089*, suggests that *slr2089* does indeed encode a functional squalene hopene cyclase, and also that if there are other enzymes in *Synechocystis* that may use squalene as a substrate, they do not consume all squalene produced under the conditions tested.

### Complementation of the Δ*shc* Strain

To confirm that the observed squalene accumulation in the Δ*shc* cells is due to the deletion in *slr2089*, we performed a complementation of the deletion in the Δ*shc* background. For this purpose, *slr2089* and an approximately 1200 bp region immediately upstream of the gene were cloned in a self-replicating vector and used to transform the Δ*shc* strain. In the resulting Δ*shc*:pPMQ*shc* strain, squalene accumulation was 0.0529±0.0031 mg OD_750_^−1^ L^−1^, and thus it was strongly reduced compared to the level in the Δ*shc* cells (Fig. 3C), showing that the introduced *shc*-gene did complement the inactivation in Δ*shc*. However, the level of squalene was not as low as in the wild type (see above and Fig. 3C). This may be due to insufficient expression from the plasmid construct.

### Inactivation of *sll0513* (*sqs*)

As described above, we identified one gene, *sll0513*, in the genome sequence of *Synechocystis,* putatively encoding squalene synthase. Since this gene is not very similar to the only cyanobacterial squalene synthase characterized so far, *sqs* in *T. elongatus* [27], we decided to investigate its function by making a deletion of this gene. We found that in the lipid extracts from the *sll0513* deletion strain, Δ*sqs,* no squalene peak could be detected by HPLC (Fig. 3C). Wild type cells did contain a low level of squalene (see above), probably present as an intermediate metabolite. The complete absence of any squalene peak in the Δ*sqs* cell extracts therefore indicates that *sll0513* really does encode squalene synthase, essential for squalene formation, in *Synechocystis*.

The results presented above show that *Synechocystis* certainly exhibits a squalene synthase activity, and this together with the conserved sequence features present in *sll0513,* the lack of squalene production in the Δ*sqs* strains, and the lack of any other obvious candidate squalene synthase genes in the *Synechocystis* genome, present a strong indication that *sll0513* does indeed encode squalene synthase in *Synechocystis*, despite the observed differences between the deduced amino acid sequence of *sll0513* and the squalene synthase of *T. elongatus* BP-1 (*tll1096*) [27]. Thus, we suggest that squalene, and hopene, formation in *Synechocystis* takes place according to the pathway presented in Fig. 1, and that *sll0513* is *sqs* in *Synechocystis*.

### Growth Characteristics of Δ*shc* Compared to Wild Type *Synechocystis*

**Figure 4:**
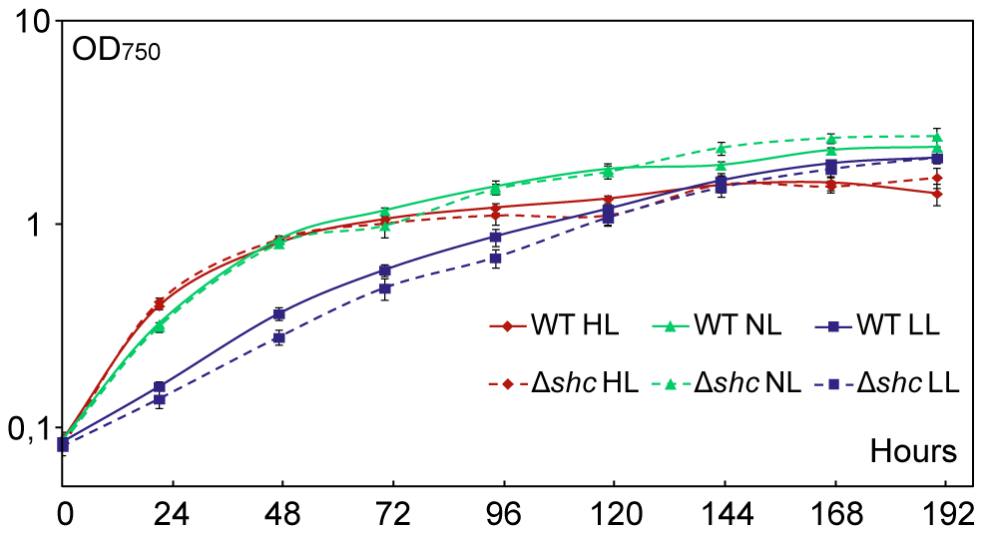
Growth curve of *Synechocystis* wild type and Δ*shc* strain under different light conditions. Wild type = solid lines; Δ*shc* = dashed lines; LL = low light (squares, 5 µmol photons m^−2^ s^−2^); NL = normal light (triangles, 50 µmol photons m^−2^ s^−2^); HL = high light (diamonds, 500 µmol photons m^−2^ s^−2^).

In order to assess the growth characteristics of the Δ*shc* strain, wild type *Synechocystis* and Δ*shc* were grown in parallel cultures under photoautotrophic growth conditions. In other organisms, it has been found that inactivating *shc* and thereby the production of hopanoids led to membrane damage [32], [33]. We therefore hypothesized that a lack of hopanoids might affect membrane systems, potentially including the thylakoid membranes, of Δ*shc*, and lead to photosynthetic growth defects. To determine the effect of Δ*shc* on the growth at different light intensities, mutant and wild type cultures were inoculated from cells grown at normal light and then grown at low light (LL, 5 µmol photons m^−2^ s^−2^), normal light (NL, 50 µmol photons m^−2^ s^−2^) and high light (HL, 500 µmol photons m^−2^ s^−2^) (Fig. 4). To quantify growth, OD_750_ was measured every 24 hours up to 192 hours. There was a marked difference in growth between different light intensities where LL had a slower initial growth but in the end reached the same OD. The difference between wild type and Δ*shc*, however, was not significant, suggesting that a loss of Shc has no impact on normal photoautotrophic growth, nor on high light induced stress tolerance under the conditions tested. Thus, if membranes in *Synechocystis* are affected by inactivating *shc*, potentially resulting in a lack of hopanoids, the effect is not so severe as to impact growth under any of the different light conditions tested. Furthermore, the accumulation of squalene was not detrimental to cell growth.

While inactivation of *shc* did not lead to sensitivity to high light stress, it is possible that other stress conditions may reveal a Δ*shc* phenotype, and we plan to address this question in future studies. It is clear from the literature that hopanoids may have different roles and be of varying importance for the growth of different organisms [32], [33], [34]. Our work in this study forms the foundation for further investigation of the role of hopanoids in cyanobacteria, which will be interesting given that they are oxygenic photosynthetic autotrophs and have a lifestyle quite different from other microorganisms where such studies have been performed so far.

### Accumulation of Squalene Depending on Light, Growth Phase

**Figure 5:**
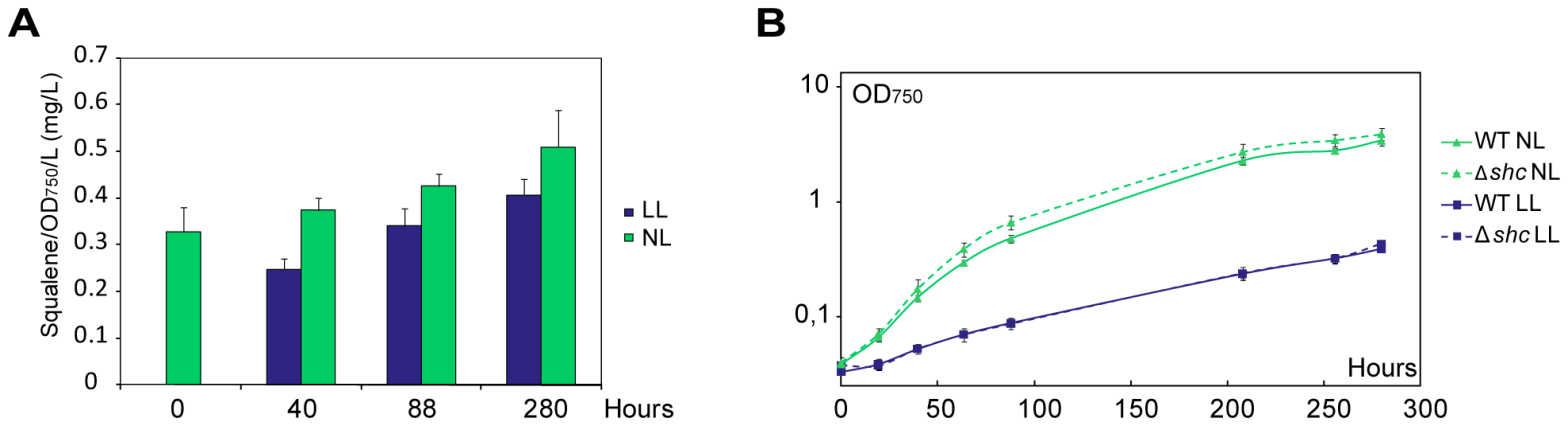
Accumulation of squalene at different growth stages and light intensities. (A) Squalene content of Δ*shc* cells grown at different light intensities and extracted at different growth stages. The mutant strain was grown under normal light, and at 0 hours low light (LL, 5 µmol photons m^−2^ s^−2^) and normal light (NL, 50 µmol photons m^−2^ s^−2^) cultures were inoculated. Squalene was then extracted at different time points to investigate the correlation between squalene production and growth phase. (B) Growth curve of Δ*shc* (dashed lines) from the same experiment, and of wild type *Synechocystis* (solid lines), using low light (squares) or normal light (triangles).

In order to investigate whether squalene production is connected to light conditions or a specific growth phase in *Synechocystis*, we collected samples of the Δ*shc* strain from cells in different growth phases and under two different light conditions, low light (LL, 5 µmol photons s^−1^ m^−2^) and normal light (NL, 50 µmol photons s^−1^ m^−2^), and examined the squalene content of the samples (see Fig. 5). The cultures were harvested in the exponential phase (40 h), late exponential phase (88 h) and in the stationary phase (280 h). Squalene content in the LL cultures at 40 hours decreased from the level in the seed culture (0 h, grown at NL prior to the start of the experiment), but then levels increased at similar rates as for the NL cultures as they grew (Fig. 5A). At each time point, cells grown under NL had higher squalene content, measured per OD_750_ and volume culture, than did LL-grown cells. This may be an effect of the lower growth rate at LL, as squalene seems to accumulate in the cells during growth. LL cultures at 280 hours reached similar OD as normal light cultures at 88 hours and also similar squalene accumulation, suggesting a correlation of squalene production and cell density. Thus, faster growing cells, leading to higher cell density, would have a higher squalene content at any given time point in a batch experiment. It should also be noted that the cultures used in the experiment had been pre-cultivated under NL and was split and moved to LL and NL conditions at the start of the experiment. The drop in squalene content in the LL grown cells after 40 h in LL presumably reflects this change in growth conditions and shift to a slower rate of growth at LL. If this change occurs because of a lower squalene production compared to growth rate, effectively diluting out the content with every cell division, or through some other mechanism is yet unclear.

## Conclusions

We have shown that it is possible to use the cyanobacterium *Synechocystis* to generate squalene, a hydrocarbon of commercial interest and a potential raw material for biofuels. We also demonstrate the first identification and functional verification of an active squalene hopene cyclase in cyanobacteria. Inactivating the *shc* gene in *Synechocystis* led to accumulation of squalene to a level about 70 times higher than in the wild type. No growth impairments were detected in the engineered strain. Furthermore, we identified a gene putatively encoding squalene synthase, and could show that inactivation of this gene abolished squalene synthesis in the cells.

The isoprenoid biosynthesis is a source of many useful compounds with wide application in biotechnology, medicine, chemistry, as food additives, and potentially as fuels. For large scale generation of useful isoprenoid compounds, it would be preferable to use photoautotrophic microorganisms such as cyanobacteria as hosts, since they can grow photosynthetically using solar energy, water and carbon dioxide from the atmosphere to generate the desired product. The squalene producing strain of *Synechocystis* generated in this study serves as an example of such a production system. Improvements of the system using metabolic engineering techniques are possible and will be addressed in future work, as well as further investigation of the native metabolism that leads to squalene production.

## Materials and Methods

### Bacterial Strains and Growth Conditions

*Escherichia coli* strain DH5α (Invitrogen) was used for subcloning and plasmid propagation. *E. coli* cells were grown at 37°C in LB medium with addition of appropriate antibiotics according to standard protocols. *Synechocystis* cells were grown at 30°C at a light intensity of about 40 µmol photons m^−2^ s^−2^, in BG11 medium [35], with addition of appropriate antibiotics for selection and growth of transformed strains. For extraction of squalene, 1 liter cultures with air bubbling were grown to an OD_750_ of ∼1 and then harvested by centrifugation, except when otherwise noted (see below).

For the growth curve experiment at three different light intensities, cultures of 25 ml were seeded from a single culture growing at normal light. Cultures were then grown with 125 rpm shaking at low light (LL) at about 5 µmol photons m^−2^ s^−2^, normal light (NL) at about 50 µmol photons m^−2^ s^−2^, or high light (HL) at about 500 µmol photons m^−2^ s^−2^. OD_750_ was measured using a 96-well plate spectrophotometer (Hidex, Turku, Finland) and converted to standard OD values by using a correlation coefficient (R^2^ = 0.9997, data not shown).

For the squalene extraction experiment at different growth stages and light intensities, 1 liter cultures were seeded and grown at LL and NL with air bubbling. Samples were removed from the culture vessels at the specified time points and squalene was extracted. All cultures were done in triplicates as well as the OD measurements.

### Generation of Genetic Constructs used in the Study

#### Inactivation of *shc*

*shc* was inactivated by replacing a 606 bp part of the gene with an antibiotic resistance cassette. Regions for homologous recombination upstream and downstream of the deleted part were amplified from *Synechocystis* genomic DNA by PCR, using primers shc_us_F (5′-tagagagaattctttgcaaacccctaaccaag-3′) and shc_us_R (5′-tagagaggatccaaattcgcagggcagtgtag-3′), and shc_ds_F (5′-tagagaggatccactagtgctatgccattcaagcctgt-3′) and shc_ds_R (5′-tagagatctagaggaatccatggtcaaaccac-3′), respectively. The upstream region was cloned in the *EcoR*V site of pBluescript SK+ (Stratagene, Agilent Technologies Inc., Santa Clara, CA, USA), resulting in plasmid pBshcU. The downstream region was then cloned in this plasmid using the *BamH*I and *Xba*I sites, to form plasmid pBshcUD. This was followed by insertion of an *npt* cassette, conferring resistance to neomycin and kanamycin, in between the upstream and downstream regions using *BamH*I, to form pBshcUND. This plasmid was used to transform *Synechocystis* cells as described elsewhere [9], [36]. After selection on plates containing 10 µg/ml kanamycin, single colonies of transformants were isolated and grown for analysis. Complete segregation of the Δ*shc* genotype was confirmed by PCR using primers shc_middle_F (5′-ttagcaaatcccgcattttt-3′) and shc_middle_R (5′-taggcccctttgcacataga-3′) in combination with shc_usR_as (5′-ctacactgccctgcgaattt-3′) and shc_dsF_as (5′-acaggcttgaatggcatagc-3′) (see Fig. 2).

Additional verification of the gene inactivation was done by sequencing. Genomic DNA was extracted and amplified using primers shc_US_for (5′-aatatgccgatggctactgg-3′) and shc_DS_rev (5′-ggaatccatggtcaaaccac-3′). Samples were then sent for sequencing (Macrogen Europe, Amsterdam, the Netherlands), using the same primers as for the amplification.

#### Complementation of the Δ*shc* strain

For complementation of the Δ*shc* strain, *shc* (*slr2089*) with its expected native promoter was amplified from *Synechocystis* genomic DNA with primers shc_comp_*Xba*I_F (5′-gagagatctagaggtggtatgttgggtatgc-3′) and shc_comp_*Pst*I_R (5′-gagagactgcagtgcaaccccctcaatcttag-3′. The resulting PCR fragment was cloned into a version of the shuttle vector pPMQAK1 [37], pPMQCmI, where the Km^R^ cassette of pPMQAK1 has been replaced by a Cm^R^ cassette (this work). The *shc* gene was cloned in to the plasmid using the *Xba*I and *Pst*I sites, to form plasmid pPMQCm*shc*. The s*hc* complementation construct was verified by sequencing and was used to transform *Synechocystis* cells as previously described [38]. After selection on plates containing 20 µg/ml chloramphenicol, single colonies of transformants were isolated and grown for analysis, resulting in strain Δs*hc*:pPMQ*shc*. The presence of *shc* in the Δ*shc*:pPMQ*shc* transformants was confirmed by PCR, comparing with wild type *Synechocystis* and Δ*shc* cells.

#### Inactivation of *sqs*

Inactivation of *sqs* (*sll0513*) was obtained by deleting 557 bp of the C-terminal end of the gene, replacing them with a chloromphenicol (Cm^R^) antibiotic resistance cassette. Four different PCR products were amplified, with 25 bp overlapping regions, to generate the plasmid construct used for the gene inactivation. The PCR products were the vector backbone of pBluescript SK+ (∼2800 bp), a region of *sqs* upstream of the deletion (524 bp), the Cm^R^ cassette (∼900 bp), and a region of the *Synechocystis* DNA downstream of the deletion (521 bp). pBluescript SK+ (Stratagene, Agilent Technologies Inc., Santa Clara, CA, USA) was used as the template for the vector backbone with the primers pBSF_new: 5′-ctccagcttttgttcccttt-3′ and pBSR: 5′-ctcactggccgtcgttttac-3′, *Synechocystis* genomic DNA was used with primers pBS_UF: 5′-acgttgtaaaacgacggccagtgaggggatcattcaggaaaagca-3′ and Cm_UR: 5′-cactcttacgtgcccgatcaactcgggggtctaatccccgaataa-3′, and Cm_DF: 5′ tggtgagaatccaagcctcgagctgcgggttattgccagttagga-3′ and pBS_DR: 5′-tcactaaagggaacaaaagctggaggtggtctgcctactggtggt-3′, for amplification of the homologous regions upstream and downstream of the deletion, and the BioBrick plasmid pSB1C3 (http://partsregistry.org/) was the template used with primers CmF: 5′-cgagttgatcgggcacgtaa-3′ and CmR: 5′-cagctcgaggcttggattct-3′ for amplification of the Cm^R^ cassette. The overlapping fragments were fused using Gibson assembly [39], resulting in plasmid pBSK+Δ*sqs*Cm^R^. The deletion construct sequence was confirmed by sequencing and used to transform *Synechocystis* cells as previously described [9], [36]. After selection on plates containing 20 µg/ml chloromphenicol, single colonies of transformants were isolated and grown for analysis. Complete segregation of the Δ*sqs* genotype was confirmed by PCR.

### DNA/RNA Extraction and RT-PCR

Nucleic acids were extracted from harvested *Synechocystis* cells using a protocol previously described by [40]. For RT-PCR, the DNA was digested from the samples using DNase I (Fermentas,Thermo Fisher Scientific Inc., Waltham, MA, USA) and the RNA was converted to cDNA using iScript™ cDNA Synthesis Kit (Biorad) according to the manufacturers’ protocol. Samples with no added reverse transcriptase were used as a negative control, and amplification of 23S using primers Syn_23S_F (5′-ctgatctccgccaagagttc-3′) and Syn_23S_R (5′-TTACCGTTGGCACGATAACA-3′) was used as a control of equal loading of the RNA (Fig. 2C).

### Extraction and Analysis of Squalene Content

Cells were harvested by centrifugation and then stored at −80°C. For the wild type/Δ*shc* comparison, sample sizes were normalized by optical density; ∼80 ml of a culture with OD_750_ 1 (∼17 mg dry weight) was used for extraction. For the light intensity experiment, ∼27 ml (∼5.3 mg dry weight) was used.

Lipid extractions of cells were based on a modified Bligh and Dyer [31] method. Pellets were resuspended with 2 ml chloroform and 4 ml methanol (both from Sigma-Aldrich, St. Louis, MO, USA). Samples were vortexed until completely resuspended and supernatants were collected by centrifugation. A phase separation was achieved of the supernatant by the addition of acetonitrile and heptane (VWR International, Radnor, PA, USA) in a ratio of 1∶1:2 (sup:ac:hep). The top, heptane phase was repeatedly washed with acetonitrile until most chlorophyll had been removed. The heptane phase was then concentrated using a rotary evaporator and a speedvac and then dissolved, first with heptane and then acetonitrile in a final ratio of 1∶20 v/v. The solution was filtered using 0.2 µm PTFE syringe filters (VWR International, Radnor, PA, USA) and then, the amount of squalene was quantified by HPLC. Using a squalene standard (Sigma-Aldrich, St. Louis, MO, USA) in 95% acetonitrile/heptane, the retention time for squalene was determined and a calibration curve was made using five different squalene concentrations between 0.5 µM and 400 µM in triplicates with a R^2^ value of 1.00. The HPLC conditions were the following: column: reverse phase C18 column (150 * 3.0 mm, 5 µm particle size, Phenomenex); column temperature: 25°C; injection volume: 20 µL; flow rate: 1.5 ml/min; detection: 190 nm; mobile phase: acetonitrile, 100% (VWR International, Radnor, PA, USA). All extractions were done on three biological replicates and two technical replicates, the results represent the mean.

### Determination of Squalene by GC-MS

Squalene analysis was performed using a Scion TQ-GC-MS/MS system chromatograph (Bruker Daltonics Inc, Billerica, MA, USA) and auto-sampler (CTC Analytics AG, Switzerland) with version 8 software (Bruker Daltonics Inc). A non-polar capillary column, DB-5MS (J & W Scientific, Folsom, CA, USA), 20 m×0.18 mm×0.18 µm film thickness, was connected to a GC. The oven temperature was programmed to 90°C for 1 minute, followed by gradual increments of 20°C/min until it reached 300°C, where it was held for 10 minutes. Squalene (Sigma-Aldrich, St. Louis, MO, USA) dissolved in hexane (200 ng/µl) was injected using a CTC injector and with a split ratio of 1∶20. The injector temperature was 250°C. Helium (99.9%) was used as the carrier gas at a flow rate of 1.00 ml/min. The mass spectra was recorded at electron energy of v70 eV and the ion source temperature was 240°C. Spectra was scanned in the range 50–500 *m/z*. Squalene was identified by comparing the retention times with that of a standard sample and its full scan mass spectrum and most instance fragments were 69.1 and 95.3. (Fig. 3A and B). Single ion monitoring (SIM) method was performed to identify squalene eluted from samples. The qualifier ions were set to 410.0 (molecular ion, M^+^), 69.1 and 95.3 *m/z*. The retention time of squalene was 7.3 min (Fig. 3A).
